# Systemic photobiomodulation in nursing professionals with chronic low back pain

**DOI:** 10.47626/1679-4435-2022-736

**Published:** 2023-02-03

**Authors:** Thais da Silva Capello Inocêncio, Rodrigo Antônio Carvalho Andraus, Rosângela Aparecida Pimenta Ferrari, Danielly Negrão Guassu Nogueira, Tatiane Tokushima, Alexandrina Aparecida Maciel Cardelli

**Affiliations:** 1 Curso de Enfermagem, Universidade Estadual de Londrina, Londrina, PR, Brazil; 2 Curso de Fisioterapia, Universidade Pitágoras, Londrina, PR, Brazil

**Keywords:** low-level light therapy, low back pain, nurse practitioners, cost, cost analysis, terapia com luz de baixa intensidade, dor lombar, profissionais de enfermagem, custo, análise de custo

## Abstract

**Introduction:**

Chronic low back pain is a frequent complaint at health care services, leading to absenteeism and high treatment costs. Photobiomodulation is a cost-effective, non-pharmacological treatment option.

**Objectives:**

To assess the cost of systemic photobiomodulation in nursing professionals with chronic low back pain.

**Methods:**

This is a cross-sectional analytical study that analyzed the absorption costing of systemic photobiomodulation in chronic low back pain and was performed in a large university hospital with 20 nursing professionals. Ten systemic photobiomodulation sessions were performed using MM Optics^®^ laser equipment at 660 nm wavelength, 100 mW power, 33 J/cm^2^ dose, for 30 minutes on the left radial artery. Direct (supplies and direct labor costs) and indirect costs (equipment and infrastructure) were measured.

**Results:**

The mean cost of photobiomodulation was R$ 25.30 ± 0.50, and the mean duration was 1,890 seconds ± 55.0. Regarding the first, fifth, and tenth sessions, labor costs were the highest (66%), followed by infrastructure (22%), supplies (9%), and the laser equipment, which presented the lowest cost (2.8%).

**Conclusions:**

Systemic photobiomodulation was shown to be a low-cost therapy when compared to other therapies. The laser equipment represented the lowest cost in the general composition.

## Abstract (pt)

**Introdução:**

A dor lombar crônica é uma queixa frequente em serviços de saúde, ocasionando absenteísmo e altos custos com o tratamento. A fotobiomodulação é uma opção de tratamento não farmacológica com bom custo-benefício.

**Objetivos:**

Aferir o custo da fotobiomodulação sistêmica em profissionais da enfermagem com dor lombar crônica.

**Métodos:**

Pesquisa transversal analítica para análise do custeio por absorção da fotobiomodulação sistêmica na dor lombar crônica, em hospital universitário de grande porte, com 20 profissionais da enfermagem. Foram realizadas 10 sessões de fotobiomodulação sistêmica com *laser* da empresa MM Optics^®^, onda de 660 nm, 100 mW de potência, dose de 33 joules/cm^2^, 30 minutos em artéria radial esquerda. Foram mensurados os custos diretos (insumos e valor da mão de obra direta do profissional) e indiretos (equipamento e infraestrutura do consultório).

**Resultados:**

O custo médio da fotobiomodulação foi de R$ 25,30±0,50, e o tempo médio foi 1.890 segundos ± 55,0. Em relação às primeira, quinta e décima sessões, o profissional apresentou o maior custo (66%), seguido da infraestrutura (22%), dos insumos (9%) e do *laser*, o qual apresentou o menor custo (2,8%).

**Conclusões:**

A fotobiomodulação sistêmica mostrou-se de baixo custo quando comparada a outras terapias. O *laser* apresentou o menor custo na composição geral.

## INTRODUCTION

Chronic low back pain (CLBP) is a frequent complaint in health care services. It affects around 80% of adults and is an important cause of leaves of absence and occupational disability, affecting work activities and quality of life and generating high treatment costs.^1-4^

Nursing is among professions that are at risk of developing low back pain, and this condition may progress to CLBP. Some activities performed by these professionals are associated with CLBP, such as postures, physical exertion, physical conditions, and characteristics of their work environment.^5^

Nonspecific CLBP lasts more than 3 months and does not have a defined cause, representing most of the cases of disorders of the lower segment of the spine, where an imbalance may happen between functional spinal load (strength required for activities of work and daily living) and potential functional capacity.^6-8^

In face of the high costs required by CLBP treatment, photobiomodulation (PBM) is an alternative, noninvasive, non-pharmacological, painless therapy that does not have side effects and has a good cost-benefit relationship for nursing professionals.^9-11^

Cost management is an important managerial tool that helps with decision-making and constitutes a strategic action for improving the provision of care.^12^

Among the various costing methods available today, we highlight absorption costing. In this method, cost estimation is divided into two stages: separation of direct and indirect costs and verification of spending with expenses, costs, and investments.^13^

PBM can be performed at the injury site or by topical stimulation on the radial artery, at the wrist region. This second modality is named intravascular laser irradiation of blood (ILIB) or systemic PBM. Among other results, this therapy has an analgesic effect.^14-16^

Considering the gap in knowledge regarding the efficacy of systemic PBM in pain mechanisms, the absence of costing studies for this therapy in the treatment of CLBP, and the incidence of this type of pain among nursing professionals, this study aimed to measure the absorption costs of systemic PBM in nursing professionals with CLBP.

## METHODS

This is a cross-sectional analytical study for verifying the absorption costing of systemic PBM for CLBP. Data collection took place in a large university hospital in the northern region of the state of Paraná between April and September 2019. The study was approved by the hospital and by the Research Ethics Committee of Universidade Estadual de Londrina (Certificate of Presentation for Ethical Appreciation 05382819100005231), opinion No. 3,107,476.

We included female nursing professionals with complaints of nonspecific CLBP and who scored ≥ 3 in a visual analogue scale (VAS). The exclusion criteria were the following: a cancer diagnosis, hypothyroidism, epilepsy or a chronic orthopaedic condition; pregnancy, individuals on sick leave or on vacation; long-term use of pain medications; and pacemakers. Women who reported occasional use of pain medications were requested to interrupt their use during laser therapy sessions.

The population consisted of 96 female workers of the following units: the Burn Center, the Emergency Room, the Female Unit, and the Surgical Unit. After invitation, 42 female workers were evaluated and 16 were excluded due to not meeting the inclusion criteria, reaching a total of 26 participants. Out of six losses, three were because of the use of pain medications during the therapy and three participants did not have the availability required for continuing the sessions. The sample of this study consisted of 20 women.

Ten consecutive, daily systemic PBM sessions were performed with each patient, with pauses on the weekends, with portable MM Optics^®^ laser equipment, manufactured in Brazil. The equipment has two diode lasers, infrared (808 nm) and visible red (660 nm), and it is registered at the National Health Surveillance Agency (ANVISA) under No. 80051420022. The equipment was set for 660 nm wavelength, 100 mW power, 33 J/cm^2^ dose, 3 J deposited energy, for 30 minutes on the left radial artery.^17^ The procedures were performed at an integrative medicine center within the research institution by the investigator, who was duly trained for performing this therapy.

We selected the absorption costing method proposed by Bertó & Beulke^18^ for estimating costs with supplies, goods, services, and diseases, considering all costs related to production according to their classification into direct and indirect costs, and fixed and variable costs.

For measuring direct costs, we collected the values paid for all supplies used and timed the mean duration of the therapy for calculating the direct labor cost (DLC). For indirect costs, we calculated the costs of laser equipment usage and clinic infrastructure, including depreciation and maintenance costs, directing the definition of apportionment units and originating proportions that were added to the direct costs for comprising the global cost. The partial direct costs (DLC and supplies) were added to the apportionment units of indirect costs (laser and infrastructure) for comprising the global cost.

For calculating DLCs, we extracted data from the Paraná state transparency database (Portal da Transparência) based on salaries from the previous 12 months. We calculated the hourly cost of a nursing practitioner who worked 40 hours a week, reaching the value of R$ 31.72.

The MMOptics^®^ laser equipment was obtained with the investigator’s own resources at the cost of 3,590.00 (one laser equipment, one plastic bracelet, and two pairs of glasses). For calculating equipment depreciation, we considered the paid value for a mean use of 48 months divided by 30 days, reaching a final daily value of R$ 2.49. Since four daily sessions were performed, the cost was established at R$ 0.62 per systemic PBM session, which was added to a 12% rate of equipment maintenance and natural wear and tear, totaling R$ 0.70.

The costs involving clinic infrastructure and supplies, such as personal protective equipment and products for disinfecting the laser equipment such as alcohol and cotton balls, were requested from the hospital’s accounting department.

Data were analyzed using SPSS software, version 20.0, being presented as means, standard deviations, and median values.

## RESULTS

The costs and durations of the first, fifth, and tenth sessions were similar. The mean cost of PBM therapy was R$ 25.30 per session, which leads to a cumulative cost per patient of R$ 250.40 for all 10 sessions (Table 1).

**Table 1:** Distribution of costs and durations of the first, fifth, and tenth therapy sessions, Londrina, state of Paraná, 2019

Characteristics	First session	Fifth session	Tenth session
Cost (R$)			
Mean ± SD	25.4 ± 0.6	25.2 ± 0.6	25.2 ± 0.3
Median (min-max)	24.6-27.0	24.0-27.1	24.8-25.9
Duration (seconds)			
Mean ± SD	1,904 ± 66.2	1,888 ± 66.9	1,878 ± 31.8
Median (min-max)	1,821-2,092	1,818-2,097	1,833-1,965

SD = standard deviation.

The first session presented a slightly higher mean cost than the others (R$ 25.40), as well as a longer duration (1,904 seconds [s]). The mean cost was R$ 25.30 ± 0.50, and the mean duration was 1,890 seconds ± 55.0. The execution of 10 sessions in 20 women totaled R$ 5,060.00 (Table 1).

**Figure 1:**
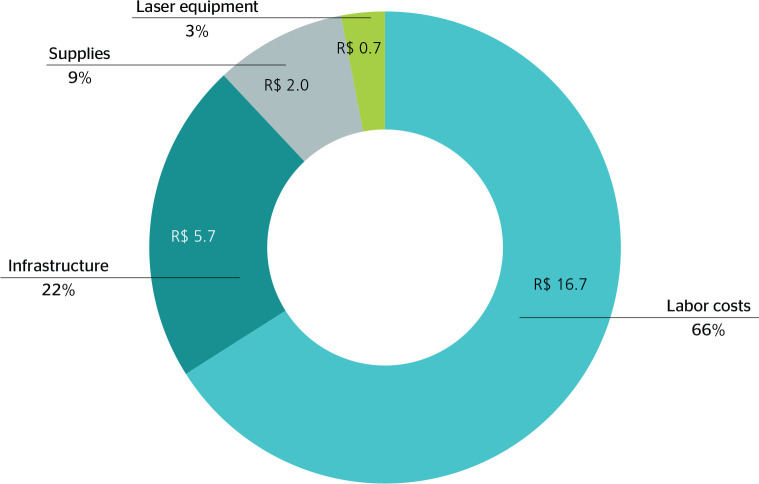
Distribution of costs and durations of the first, fifth, and tenth therapy sessions, Londrina, state of Paraná, 2019.

When performing the first, fifth, and tenth laser sessions and considering direct costs per patient, labor costs were the highest: R$ 16.70 (66%), followed by supplies, at R$ 2.00 (9%). As for indirect costs, infrastructure represented a cost of R$ 5.70 (22%) and the laser equipment had the lowest general cost: R$ 0.70 (3%). Direct costs represented 75% of the total amount, while indirect costs accounted for 25% (Figure 1).

## DISCUSSION

More important than knowing the cost of this therapy is understanding the cost apportionment so that managers can prioritize managerial actions with items that are more strongly represented in the composition of costs, possibly incorporating to his or her decision-making what, how, and where these resources are spent.

In the perspective of health care management, the best cost analysis identified in this study lies in the low cost of the laser equipment in the total composition of a PBM session. A study performed with patients with CLBP compared the cost-benefit relationships of electroacupuncture (EA) and nonsteroidal anti-inflammatory drugs (NSAIDs). The total cost of EA per patient was US$ 461.50, and that for NSAIDs was US$ 497.80,^19^ which corresponded to R$ 1,954.00 and R$ 2,107.60, respectively. These values are around 77 and 83 times higher than that for systemic PBM, respectively.

CLBP is a condition that requires substantial economic resources from the health care system; annual costs are estimated at US$ 100 billion. This is why scientific evidence is necessary to guide management and avoid unnecessary procedures.^20,21^

A study verified that, in the 6 previous months, direct costs (use of health care services and medications) and indirect costs (loss of productivity) with CLBP totaled US$ 15.49 (around R$ 65.63), and medications represented 60% of the direct costs. Indirect costs represented 31%. Participants missed around 12 workdays and 49 days of school/housework.^22^ The expenses with CLBP are too high when compared to those for PBM, which are 2,594 times smaller than the costs reported by the aforementioned study.

Considering these costs, it is possible to recognize the importance of incorporating systemic PBM in the treatment of CLBP; it represents non-pharmacological approach that, among other effects, has an analgesic action and contributes to decreasing costs allocated to the treatment of CLBP. It is worth highlighting that this therapy is not funded by the Unified Health Care (SUS) in Brazil.^9,10,16^

It is important to think of a therapeutic alternative because nursing professionals are exposed to occupational risks on a daily basis, and these can compromise their physical and mental health. Low back pain is among the main diseases that can affect nursing practitioners due to ergonomic risks to which they are exposed. This way, health management should incorporate strategies.^23^

This therapy can be performed at the workplace, which is convenient for employees and employers because workers do not need to be absent for long periods. Although the employees are not productive during therapy sessions, PBM treatment can decrease absenteeism rates since CLBP is an important cause of leaves of absence.^2,4^

Between 2012 and 2016, the mean number of sick leave days due to low back pain was around 80 to 100 days per year, and the total costs related to leaves of absence were around 59 million days. In total, 668,206 people were on leave, generating costs to the National Social Security Institute (INSS). This way, this therapy could also contribute to decrease social security expenses with paid leaves.^24^

Apart from absenteeism, medical costs are increased by CLBP treatments, as data have demonstrated. Pain affects worker productivity and decreases quality of life, characterizing a public health problem due to its high prevalence. Considering these factors, incorporating PBM could be positive to quality of life at work, in addition to minimizing future expenses with treatment and compensations.^5^

The variation identified in the first session is related to the time spent by nursing professionals with the initial guidance about the study and PBM, being directly related to the DLC calculation, which is the cost of the timed work hour.

Although the cost of the laser equipment was the highest when compared to the other costs, it represented the smallest value in the therapy. Moreover, it is user-friendly and should be operated by a trained professional.

The practical application of this study encompasses two important aspects: the first is that other studies seeking alternative non-pharmacological treatments for CLBP will be able to incorporate cost estimates into comparative research; the second is that managers can incorporate the newly estimated costs into their decision-making process for establishing occupational health policies aimed at nursing professionals.

New studies that assess the impact of costs in the society’s perspective could be developed after improvements to national INSS databases.

A limiting factor of this research was the reduced size of our sample, because many women presented exclusion criteria and their availability was limited even though the duration of the therapy was short; in addition, long-term effects/therapy maintenance were not verified.

## CONCLUSIONS

Systemic PBM was shown to be a low-cost approach when compared to other treatments for nonspecific CLBP such as the use of medications. As to cost compositions, labor costs presented the highest value, and although the laser equipment had a significant cost, it represented the variable with the lowest cost. Considering these results, the use of this therapy could improve the quality of work activity, decreasing leaves of absence and consequently contributing to decrease expenses with paid leaves.
